# Cerebrospinal fluid protein biomarkers are associated with response to multiagent intraventricular chemotherapy in patients with CNS lymphoma

**DOI:** 10.1093/noajnl/vdaf046

**Published:** 2025-02-25

**Authors:** Aastha Aastha, Hannah Wilding, Nicholas Mikolajewicz, Shahbaz Khan, Vladimir Ignatchenko, Leonardo Jose Monteiro De Macedo Filho, Debarati Bhanja, Gabriela Remite-Berthet, Madison Heebner, Michael Glantz, Alireza Mansouri, Thomas Kislinger

**Affiliations:** Princess Margaret Cancer Centre, University Health Network, Toronto, Ontario, Canada; Department of Medical Biophysics, University of Toronto, Toronto, Ontario, Canada; Department of Neurosurgery, Penn State Milton S. Hershey Medical Center, Hershey, Pennsylvania; Division of Neurosurgery, Department of Surgery, University of Toronto, Toronto, Ontario, Canada; Peter Gilgan Centre for Research and Learning, Hospital for Sick Children, Toronto, Ontario, Canada; Princess Margaret Cancer Centre, University Health Network, Toronto, Ontario, Canada; Princess Margaret Cancer Centre, University Health Network, Toronto, Ontario, Canada; Department of Neurosurgery, Penn State Milton S. Hershey Medical Center, Hershey, Pennsylvania; Department of Neurosurgery, Penn State Milton S. Hershey Medical Center, Hershey, Pennsylvania; Department of Neurosurgery, Penn State Milton S. Hershey Medical Center, Hershey, Pennsylvania; Department of Neurosurgery, Penn State Milton S. Hershey Medical Center, Hershey, Pennsylvania; Department of Neurosurgery and Oncology, Penn State Milton S. Hershey Medical Center, Hershey, Pennsylvania; Department of Neurosurgery, Penn State Milton S. Hershey Medical Center, Hershey, Pennsylvania; Princess Margaret Cancer Centre, University Health Network, Toronto, Ontario, Canada; Department of Medical Biophysics, University of Toronto, Toronto, Ontario, Canada

**Keywords:** cerebrospinal fluid, CNS lymphoma, proteomics

## Abstract

**Background:**

Central nervous system lymphoma (CNSL), is a rare subtype of non-Hodgkin lymphoma, primarily affecting the brain and spinal cord. Most therapeutic systemic agents have limited penetration of the blood-brain and blood-cerebrospinal fluid (CSF) barrier, with the latter potentially promoting a treatment “sanctuary” for cancer cells. Evaluation of occult disease, particularly in the CSF, is challenging. In limited clinical experience, the addition of multiagent intraventricular chemotherapy (MAIVC), delivered through intracranially implanted CSF reservoirs, to systemic therapy has demonstrated encouraging outcomes, enhancing both progression-free survival and overall survival. However, given the potential morbidity associated with MAIVC, identification of minimally invasive biomarkers for guiding patient selection and management is necessary. Leveraging the longitudinal, large volume of CSF, the objective of this study was to identify CSF-based proteomic biomarkers that can serve as reliable indicators of CSF clearance in response to MAIVC and CNSL treatment outcome.

**Methods:**

One hundred fifteen CSF samples from 59 CNSL patients receiving MAIVC were profiled using a high-throughput protocol coupled with mass-spectrometry that only requires 30 μL of CSF.

**Results:**

More than 2000 unique proteins were detected using shotgun proteomics. Cerebrospinal fluid proteomics revealed key proteins (SGCE, LCP1, AGRN, OLFML3, and HRSP12) distinguishing early from never responders to MAIVC, with area under the receiver operating characteristic (AUROC) 0.86 (95% CI: 0.696-1). By integrating tumor volume from brain MRI scans with proteomic data, we identified potential intraventricular tumor burden markers for CNSL management, in particular LCP1.

**Conclusions:**

The study identified CSF-based proteomic biomarkers, particularly LCP1, that can classify MAIVC response and indicate tumor burden in CNSL patients.

**Key Points**

- CSF-based biomarkers can aid CNSL diagnosis, prognosis, and treatment monitoring.
- LCP1 identified as potential biomarker of CSF clearance in response to MAIVC and tumor burden in CNSL.

**Importance of the Study**

Our study underscores the potential of CSF proteomics as a tool for managing CNSL and enhances our understanding of the molecular underpinnings associated with treatment response and disease progression.

Central nervous system lymphoma (CNSL) is broadly classified as primary CNSL (PCNSL) or secondary CNSL (SCNSL). Primary CNSL is confined to the CNS (brain, spinal cord, leptomeninges, or eyes) at the time of diagnosis and lack systemic involvement, while SCNSL arises outside of the nervous system, and spreads to the CNS.^1–5^

High-dose methotrexate-based systemic therapy, followed by autologous stem cell transplantation (ASCT) where feasible, is the cornerstone of management for CNSL.^6–8^ The latter is either part of the consolidation phase of therapy or for patients presenting with relapse who have a good performance status. Though the prognosis of PCNSL is superior to SCNSL, with exquisite responses to initial therapy, relapse is common, and overall responses are disappointing. More aggressive treatment strategies using multiagent systemic chemotherapies have been more effective but at the cost of increased toxicity.^9,10^ Other consolidation regimens involving whole brain radiation therapy (WBRT) are associated with a greater risk of debilitating neurotoxicity and cognitive decline and tend to be now reserved for individuals who are otherwise not candidates for other therapies or clinical trials.^11^

Current diagnostic and monitoring strategies for CNSL rely on magnetic resonance imaging (MRI) and cerebrospinal fluid (CSF) cytology ± flow cytometry.^12^ While MRI provides high-resolution anatomical imaging, it lacks the specificity to distinguish CNSL from other pathologies, such as high-grade gliomas or metastatic lesions.^12–14^ This limitation can result in false positive diagnoses or delayed treatment. Furthermore, MRI is unable to evaluate for residual disease isolated to the CSF. While evaluation of the CSF through cytology and/or flow cytometry can provide valuable information, the sensitivity of current assay methods is limited.^13,15^ Consequently, relying on either imaging or cytology may fail to detect minimal residual disease (MRD) or subtle changes indicative of CNSL progression. In addition, no cytologic, flow cytometric, or MRI characteristics have proved consistently valuable in optimizing treatment selection or providing an early indication of treatment response. Thus, while MRI and CSF cytology are complementary techniques and are currently widely implemented in the clinic, their combined use still results in diagnostic, prognostic, and predictive uncertainty. This underscores the need for additional sensitive and specific biomarkers for CNSL to improve patient outcomes.

Confirmation of MRD, both within the brain parenchyma and in the CSF, is critical for therapeutic decision-making. With more novel therapies, such as chimeric antigen receptor T-cell (CAR T Cell) therapies which have seen relative success with hematological malignancies, there has been traditional hesitancy to initiate treatment in those with CNS positive disease due to concerns regarding increased risk of neurotoxicity.^16^ Evaluation of MRD is critical for interpretation of clinical trial data as well. Two clinical trials comparing WBRT to autologous stem cell transplant (ASCT) for consolidation (PRECIS and IELSG32) demonstrated contradictory data,^17,18^ with one hypothesis being variable MRD across enrolled patients. Unlike other hematological malignancies, disease management in PCNSL is not informed by MRD. Cerebrospinal fluid analyses have identified promising diagnostic biomarkers, including IL10 and *MYD88* mutations; however, longitudinal studies evaluating whether these biomarkers can be leveraged to detect imaging-occult relapse or residual disease are lacking.

Drawing from dramatic improvements in leukemia survival and based on the notion that the CSF is a sanctuary for residual cancer cells, some have advocated for the addition of intraventricular chemotherapy to the systemic regimen for managing CNS lymphoma as well.^19,20^ Specifically, multiagent intraventricular chemotherapy (MAIVC), delivered through an intracranial CSF reservoir, has been proposed as a strategy to not only enhance drug concentration at the tumor site and reduce systemic side effects but also for disease monitoring.^19,21^ Preliminary studies using MAIVC have shown promising improvements in overall survival for certain patient subpopulations, though clinical response to MAIVC remains variable.^19,22,23^ While systemic toxicities are rare with MAIVC, headaches, seizures, neurocognitive deficits, and catheter-related complications can occur.^24^ Given these potential toxicities and variable responses, there is a need to distinguish patients who would benefit most from MAIVC from those who might be more appropriate candidates for proton beam craniospinal irradiation, novel protocol-based therapies,^25^ or clinical trials (eg, lenalomide maintenance for PCNSL patients not eligible for ASCT NCT05260619, TEDDI-R trial for SCNSL NCT03964090, selenixor + methotrexate + rituximab in relapsed/refractory CNSL NCT05698147, or F520 for relapsed/refractory CNSL NCT04457869).

Molecular profiling of CSF is a promising strategy to diagnose and monitor intracranial pathologies. The CSF represents a snapshot of the intracranial milieu, meaning that microenvironmental changes along with tumor-specific molecular signatures can be detected in CSF collected by lumbar puncture or ventricular reservoir tap. Recent work from our group^26^ and others^27^ has shown that the CSF proteome can reliably differentiate malignant from nonmalignant disease, including in CNSL patients.

We performed mass spectrometry-based proteomic analysis of 115 CSF samples collected from patients with CNSL in 59 patients. The aims of our analyses were 2-fold: (i) to identify biomarkers associated with CSF clearance in response to MAIVC and (ii) to identify biomarkers of tumor burden within the CSF. For each subset of markers, we developed and internally validated classification models. In addition to demonstrating the feasibility of using low-input CSF proteomics in the management of CNSL, we outline candidate biomarkers associated with clearance of disease from the CSF following MAIVC and useful for monitoring tumor burden within the CSF.

## Methods

### Ethics Statement

Single-institution data from the Penn State Health electronic medical record were utilized to perform the retrospective chart review of CNSL patients treated from 2016 to 2022. Institutional Review Board approval was provided to the Penn State Neuroscience Biorespository by Penn State University (IRB #2914) and by the Research Ethics Board of the University Health Network, Toronto, Canada (23-5760).

### Data Collection

A list of patients diagnosed with PCNSL or SCNSL and treated by Penn State Health neuro-oncology providers was compiled. Inclusion criteria included age >18, diagnosis of PCNSL or SCNSL, and receiving MAIVC under the direction of a medical neuro-oncologist at Penn State Hershey Medical Center. Within these methods, MAIVC refers to intraventricular therapy pertaining specifically to CNSL, and not broadly to intraventricular treatments for other cancers or brain tumors. Dates of CSF sample collection ranged from 29 February 2016 to 16 March 2022. Cerebrospinal fluid was available for deposit in our CSF biorepository from all participating patients and from each treatment cycle, in accordance with our IRB-approved biorepository protocol. Clinical data were collected via chart review on all patients from time of their first diagnosis of CNSL. In addition to age and sex, the following disease data were collected: CNSL type (PCNSL, SCNSL), method of CNSL diagnosis (imaging, brain biopsy, CSF cytology), dates of CSF sample collections, CSF cytology and flow cytometry at every CSF sampling, CNSL treatment history (surgery, biopsy, chemotherapy, and radiation), MRI brain/spine imaging (tumor volume), presence of leptomeningeal disease (LMD) on imaging, intraventricular chemotherapy treatment regimen, and overall survival. Brain MRI images performed closest to the date of CSF sample were recorded with their corresponding CSF sample. If patients had an initial negative brain MRI (ie, their nervous system disease was confined to the CSF), serial brain MRI’s were not performed unless clinically indicated, or if their disease proved refractory to therapy. Such patients were classified as having no “measurable disease” in the CNS for the purposes of volumetric tumor burden measurements.

### MAIVC Regimen

At time of CNSL diagnosis, patients underwent image-guided endoscopic insertion of an Ommaya reservoir. Utilizing a protocol specific to our institution (Penn State) (Supplementary Table S1), MAIVC is considered as an option for any patient with suspected or confirmed LMD from solid tumor malignancies and all cases of CNSL. Patients then received 6 cycles of weekly induction MAIVC therapy consisting of thiotepa 15 mg plus gemcitabine 5 mg alternating weekly with cytarabine 100 mg plus etoposide 2 mg (ie, thiotepa and gemcitabine were administered 1 week, followed by cytarabine and etoposide the next week, for 6 weeks). Beginning in May 2021, 10-mg vancomycin was added to each administration of intraventricular chemotherapy to optimize infection prophylaxis. If CSF cytology at the end of induction was negative, patients proceeded to maintenance therapy which consisted of an addition 6 cycles of therapy (2 cycles every 2 weeks, then 2 cycles every 3 weeks, and finally 2 cycles every 4 weeks) using the same agents and doses in alternating sequence—thiotepa plus gemcitabine for cycles 7, 9, and 11, and etoposide plus cytarabine for cycles 8, 10, and 12. If cytologic response to therapy was incomplete, or if patients failed to tolerate one of the initial chemotherapeutic agents, topotecan 0.4 mg, rituximab 25 mg, or methotrexate 15 mg was substituted, and 2-4 additional weekly cycles of intraventricular chemotherapy were administered before proceeding to maintenance therapy. Cerebrospinal fluid cytology was evaluated at the time of each treatment.

At the end of cycle 12 (6 induction cycles + 6 maintenance cycles), patients with a persistently positive CSF cytology were continued on the maintenance schedule of MAIVC if they remained neurologically stable (Supplementary Figure S1A). To monitor for relapse, patients with negative CSF cytology were placed on a standard CSF surveillance regimen consisting of large volume (typically 15-30 mL) diagnostic ventricular reservoir taps at 1, 2, 3, 6, 9, and 12 months, and then every 4 months for an additional 2 years. At the time of each tap, CSF not used for clinically necessary studies (typically 3-10 mL) was placed in the Penn State Neuroscience Biorespository, according to our IRB-approved protocol.

In addition to MAIVC, high-dose methotrexate at 8 g/m² and Rituximab at 375mg/m² were used for systemic chemotherapy. A dose reduction protocol was used for patients with reduced renal clearance, as per established clinical protocols.

### Proteomic Profiling

#### Sample preparation.—

Sample preparation was performed as described previously.^26,28^ Briefly, for each CSF sample, protein concentrations were estimated using BCA assay (Pierce). Cerebrospinal fluid volumes containing 50 μg of protein was used for sample processing, and 2 pmol of *Saccharomyces cerevisiae* invertase 2 (SUC2) was spiked in each sample as a processing control. Proteins in each sample were reduced with 5-mM dithiothreitol at 60 °C for 30 min, followed by alkylation with 25-mM iodoacetamide in the dark for 30 min at room temperature. For protein purification, an adapted MStern technique^28^ was used, where samples were passed through a polyvinylidene fluoride 96-well MStern plate (Millipore) using a vacuum suction manifold (Millipore). To facilitate binding, the membrane was equilibrated with 100 µL of 70% ethanol, followed by washing twice with 100-mM ammonium bicarbonate (ABC). The adsorbed proteins were rinsed with 100-mM ammonium bicarbonate (pH 8) and subsequently digested at 37 °C for 4 h using 50 µL of a digestion buffer (5% acetonitrile, 100-mM ammonium bicarbonate, and 1-mM calcium chloride), with 2-µg mass-spectrometry grade Trypsin-LysC (Promega). The resulting peptides were eluted from the membrane using 50% acetonitrile, followed by lyophilization with a vacuum concentrator and desalting using C18 stage tips (3M Empore). The desalted peptides were again lyophilized and resuspended in 0.1% formic acid in liquid chromatography (LC)-MS grade water. Peptide concentration was determined using a Nanodrop 2000 (Thermo Scientific).

#### Data acquisition.—

Synthetic indexed retention time (iRT) (Biognosys) standard peptides were spiked into each sample at a ratio of 1:10. A volume equivalent to 1 µg of peptides along with iRT was loaded onto a 2-cm PepMap Acclaim trap column (Thermo Scientific) using Easy1000 nanoLC (Thermo Scientific). The peptides were separated over a 2-h reversed-phase gradient using a 50-cm EasySpray ES903 analytical C18 column (Thermo Scientific) coupled to a QExactive HF Orbitrap mass spectrometer (Thermo Scientific). The data were acquired in a top-20 data dependent mode. MS1 spectra were acquired at a resolution of 120 000, with an automatic gain control (AGC) target of 1e6 ions and a maximum fill time of 40 ms. The MS2 data were acquired at a resolution of 30 000, with an AGC target of 2e5 ions and a maximum fill time of 55 ms. Dynamic exclusion was set for 20 s, and the normalized collision energy was adjusted to 27%.

#### Mass spectrometry raw data analysis.—

Raw files were analyzed using MaxQuant (version 1.6.3.3) and searched against a UniProt complete human protein sequence database (v2022_04) merged with yeast invertase (SUC2) and synthetic iRT sequence peptides.^29^ For all the searches, 2 missed cleavages were permitted and carbamidomethyl modification was specified as a fixed modification along with variable oxidation of methionine and variable acetylation of the N-terminus of protein. Peptide false discovery rate (FDR) was regulated using a target-decoy strategy involving reversed sequences, with a set threshold of 1% at the site, peptide, and protein levels. Label free quantification (LFQ) intensities were used, and proteins detected with at least 2 peptides were kept for downstream analysis. Missing LFQ values for proteins were imputed with median-adjusted intensity-based absolute quantification values.^30^ Log_2_ transformed intensities were used for subsequent analyses unless otherwise stated. Two samples were removed from further analysis as the CSF was collected by lumbar puncture.

### Clinical Assessment Criteria

#### Diagnosis of CNS lymphoma.—

Our approach to the evaluation of patients with CNS lymphoma followed established algorithms.

In patients presenting with clinical symptoms, suspicious brain MRI findings, and absence of known history of extra-CNS lymphoma, evaluation of the CSF for cell count, protein, cytology, and flow cytometry was conducted. Given the low sensitivity of these standard analyses, the diagnosis was often supplemented by stereotactic brain biopsy, which was used to rule out other brain pathologies as well. As vitreoretinal lymphoma is a variant of PCNSL, a vitrectomy, vitreous fluid cytology, or vitreous biopsy demonstrating malignant cells was sufficient for diagnosis (Table 1). Upon confirmation of CNS lymphoma, additional staging procedures (eg, ophthalmological evaluation, testicular ultrasound, and PET imaging) were conducted to rule out SCNSL.

**Table 1.** Summary of Retrospective CNSL Cohort

Characteristic	PCNSL (*N* = 21)	SCNSL (*N* = 38)
**Demographics**		
Age at diagnosis (years; mean ± SD)	67.0 ± 11.7	61.7 ± 14.9
Female [*n* (%)]	9 (43)	12 (32)
Male [*n* (%)]	12 (57)	26 (68)
**Single Most Definitive CNSL Diagnostic Method**		
Clinical Judgement (with imaging) [*n* (%)]	0 (0)	12 (32)
CSF^*^ [*n* (%)]	0 (0)	22 (58)
Brain Pathology (From Surgery or Biopsy) [*n* (%)]	19 (90)	4 (10)
Vitreous Diagnosis [*n* (%)]	2 (10)	0 (0)
**Response Classification**		
Early Responder (*n*)	12	19
Intermediate Responder (*n*)	4	7
Late Responder (*n*)	1	0
Never Responder (*n*)	3	10
Withdrew (*n*)	1	2
**CNSL Treatment History**		
Surgery [*n* (%)]	4 (19)	4 (11)
Radiation [*n* (%)]	4 (19)	7 (18)
Systemic Chemotherapy [*n* (%)]	21 (100)	38 (100)
**MRI Brain Classification at Initial CSF Collection**		
Abnormal (eg, lesion, hydrocephalus, FLAIR change) [*n* (%)]	15 (71)	22 (58)
Normal [*n* (%)]	6 (29)	16 (42)
**MRI Brain Classification at Post CSF collection**		
Abnormal (eg, lesion, hydrocephalus, FLAIR change) [*n* (%)]	11 (52)	16 (42)
Normal [*n* (%)]	10 (48)	22 (58)
**Volume of Measurable Disease (mm** ^**3**^ **)**		
“Early” Responders at “Baseline” (8)	9318	9188
Early Responders Post	564	1920
“Never” Responders at “Baseline” (7)	34506	36607
Never Responders Post	8806	6398
**CSF Cytology Classification at Initial CSF Collection**		
Atypical/Negative [*n* (%)]	19 (90)	32 (84)
Positive [*n* (%)]	2 (10)	6 (16)
**CSF Cytology Classification at Post CSF Collection**		
Atypical/Negative [*n* (%)]	21 (100)	38 (100)
Positive [*n* (%)]	0 (0)	0 (0)
**CSF Flow Cytometry**		
Had flow cytometry performed	18 (86)	34 (89)
Instance of positive flow cytometry	3 (14)	16 (42)
**Intraventricular Chemotherapy Treatment**		
Number of Cycles (median (range)) ^**^	9 (2–22)	8 (3–23)

^*^Patients were considered to have diagnostic CSF and hence able to start multiagent intraventricular chemotherapy (MAIVC) if their CSF cytology showed malignant or atypical suspicious cells or if flow cytometry contained diagnostic markers. ^**^Median number of MAIVC cycles received for each patient at time of their post sample in our dataset.

In patients with a known history of extra-CNS lymphoma, concerning clinical or craniospinal imaging findings were further confirmed with evaluation of CSF and brain biopsy. In rare cases, patients with lymphoma subtypes known to be at high risk of CNS dissemination (eg, double hit lymphoma) were treated empirically. Such high-risk patients who were diagnosed with SCNSL on imaging and treated empirically were considered to have a “clinical” diagnosis of CNSL (Table 1). In situations of a “clinical” diagnosis of CNSL, it should be understood that imaging significantly informed clinical judgement and hence the decision to treat. In contrast to the “CSF Tumor burden assessment” described below, patients were considered to have a “CSF” diagnosis of CNSL if their CSF cytology showed pathologist-confirmed definitively malignant cells OR pathologist-confirmed “atypical cells” suspicious for CNSL. Given the morbidity of CNSL, our threshold for diagnosing and initiating treatment for CNSL was lower than that for quantifying CSF tumor burden.

While multiple methods of diagnosis were utilized to investigate and confirm CNSL diagnoses in our patient cohort, the single most definitive method of CNSL diagnosis (CSF, brain pathology, clinical judgement [with imaging], vitreous diagnosis) for each patient is displayed in Table 1.

#### Timing of sample collection.—

Two longitudinal samples were analyzed for each patient. Due to limited CSF volume remaining after clinical testing, samples were pooled according to their stage in the treatment, according to the following criteria:

1. “Baseline” (*n* = 43): First CSF collection obtained within the first 3 cycles.
2. “Intermediate” (*n* = 12): First CSF collection obtained during MAIVC cycles 4-12.
3. “Post” (*n* = 15): Follow-up samples retrieved after MAIVC cycle 12.

#### Response classification.—

To identify proteins associated with CSF disease clearance in response to MAIVC, “Baseline” CSF samples were retrospectively stratified by the patient’s response to treatment. Treatment responsiveness was classified by the number of MAIVC cycles required to achieve a negative CSF cytology, whether positive for malignant or atypical cells at baseline, and the durability of response. Specifically:

1. “Early” responders (*n* = 23): (i) Clearing of CSF cytology achieved within 6 MAIVC cycles and (ii) CSF cytology remains negative until end of treatment.
2. “Intermediate” responders (*n* = 5): (i) Negative CSF cytology achieved during MAIVC cycles 7-12, and (ii) CSF cytology remains negative in 2 or more subsequent consecutive samples.
3. “Late” responders (*n* = 1): (i) Negative CSF cytology achieved after MAIVC cycle 12, and (ii) CSF cytology remains negative for 2 or more subsequent consecutive samples.
4. “Never” responders (*n* = 11): (i) Negative CSF cytology never achieved, or (ii) salvage treatment was required (eg, CNS surgery or radiation) during MAIVC treatment.
5. Withdrew (*n* = 3): Patients who withdrew before having received 12 cycles of MAIVC for reasons other than death or salvage therapy.

#### CSF tumor burden assessment.—

To identify proteomic tumor burden markers, our evaluation of CSF positivity was modified. Specifically, we implemented a stringent set of criteria and analytical steps to increase the fidelity of our results within the limits of the retrospective nature of this study. For the purposes of tumor burden evaluation, our goal was to focus on samples that had clear involvement of CSF and brain parenchyma. Therefore, among samples identified as “Baseline,” we limited our analysis to only those with “measurable disease” on MRI, defined as at least one distinct lesion with tumor diameter of 1 cm on single voxel on 1-mm slice thickness contrast-enhanced *T*_1_-weighted CNS neuroaxis imaging^31^ or confirmed “malignant cells” on CSF cytology, as determined by a board-certified cytopathologist. All other cytology classifications, including those deemed “atypical” by a pathologist, were not included for this analysis to maintain rigor. Samples meeting either the measurable disease or malignant CSF cytology criterion were classified as “Evaluable tumor burden,” while those that did not meet either criterion were classified as “No Evaluable tumor burden.” This is different from our approach to the evaluation of response noted above, wherein “atypical cells” and “negative for malignancy” were both acceptable cytology findings for the evaluation of CSF clearance.

### Bioinformatic Analysis

#### Data preprocessing.—

For all analyses, the mass-spec intensity matrix (protein × patients) was filtered to retain proteins present in at least 60% within each comparison group (disease type [PCNSL vs SCNSL], longitudinal stage [“Baseline” vs “Post”], response status [“Early” vs “Never”], and tumor burden [“Evaluable” vs “No evaluable”]). No imputation was performed.

#### Differentially expressed proteins.—

Differentially expressed protein (DEP) analysis was performed between each comparison using unpaired Student’s *t*-test (t.test function, *stats* v4.3.0 R package).

#### Functional annotation.—

Gene set enrichment analysis was performed using proteins ranked by their log_2_-fold change (log_2_FC) values, calculated by DEP analysis between each comparison group. Gene ontology Biological Processes (BP) was the reference standard, with gene sets constrained to a minimum of 25 and a maximum of 200.

#### Survival analysis.—

Univariate Cox proportional hazards regression models (coxph function, *survival* v3.5-7 R package) were used to determine the association between CSF protein intensity and survival. Kaplan-Meier analysis was performed to visualize survival associations (survfit2 function, *ggsurvfit* v1.0.0 R package).

#### MAIVC response classification model.—

To identify proteins that distinguish between “Early” and “Never” responders to MAIVC treatment, we performed regularization-based feature selection and developed a classification model. Initially, 785 proteins were considered (detected in >60% of samples in each response group) and missing values were replaced with zero. Informative proteins were identified using L1-regulatorized logistic regression (glmnet function, *glmnet* v 4.1-8 R package) with leave-one-out cross validation (LOOCV, *n* = 34 folds). Proteins selected in at least 3/34 LOOCV folds were then filtered by significance, as determined by DEP analysis between “Early” and “Never” responders (*p* < .05). The selected proteins (*n* = 5) were used to train 3 independent algorithms: logistic regression (glm function, *stats* v4.3.0), naïve bayes (naiveBayes function, *e1071* v1.7–14), and random forest (randomForest function, *randomForest*, v4.7-1.1) and internally validated with leave-one-out-cross validation. Model performance was determined by area under the receiver operating characteristic (ROC) curve analysis (roc function, *pROC* v1.18.5 R package).

#### CSF tumor burden biomarkers.—

To identify biomarkers associated with CSF tumor burden, we performed DEP analysis (log_2_FC > 0.7) for the following comparisons:

1. “Baseline” vs “Post”: higher tumor burden expected at baseline prior to treatment.
2. “Evaluable tumor burden” vs “No evaluable tumor burden”: higher tumor burden expected in evaluable disease.

Furthermore, to ensure our analyses were robust, we repeated the DEP analysis for the longitudinal analysis (“Baseline” vs “Post”) using matched and unmatched samples. Proteins consistently identified across all comparisons were evaluated in external cohorts. Where possible, the Spearman correlation between selected protein and tumor volume (determined by MRI imaging) was determined.

#### Data visualization.—

Unless otherwise stated, all plots were generated using R programming language (v4.3.0) with *ggplot2* R package (v3.4.4). Venn diagrams were generated using *ggVenn* R package (0.1.10). Heatmaps were generated using *ComplexHeatmap* R package (version 2.18.0).

#### Statistical analysis.—

Significance was reported as *p*-value < .05*, < .01**, or < .001***, unless otherwise specified. Data normality was assessed visually. Unless otherwise specified, comparisons were performed using unpaired Student’s *t*-tests and correlations were determined using the Pearson correlation coefficient.

## Results

### Proteomic Profiling of CSF Collected from CNSL Patients Treated by MAIVC

**Figure 1.**
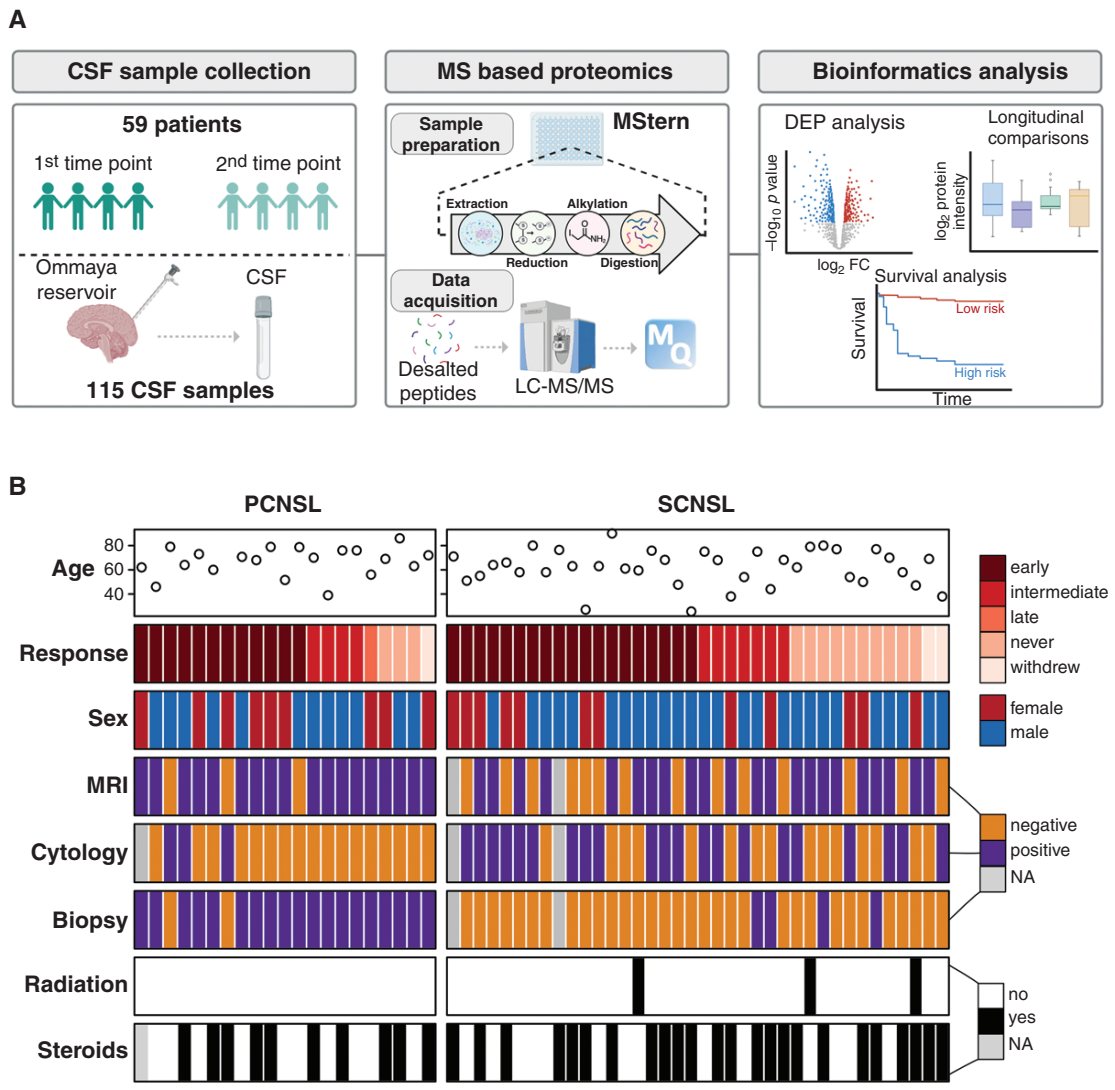
Proteomic workflow for identification of CSF prognostic biomarkers to MAIVC therapy. (A) Schematic representation of the workflow. Created with BioRender.com. (B) Heatmap representing clinical parameters of CNSL patients undergoing MAIVC therapy. Data shown correspond to the first sample collected from each patient. CSF: Cerebrospinal fluid; MAIVC: multiagent intraventricular chemotherapy.

We identified a retrospective cohort of 59 CNSL patients (21 PCNSL and 38 SCNSL) who underwent MAIVC treatment at Penn State Health (Figure 1A). The median age of the cohort was 65 years old (range: 25.6-90 years) and 63.2% were males (Table 1). For each patient, at least 2 CSF samples were collected at different stages of treatment (Figure 1B).

Using liquid chromatograpy with tandem mass spectrometry (LC-MS/MS), we performed proteomic profiling of the CSF samples and detected a total of 2470 proteins. After applying the appropriate quality filters (proteins detected with at least two peptides), we retained 2059 unique proteins, corresponding to a median of 1006 proteins per CSF sample (Supplementary Figure S1B). Among these, 86.6% (1745/2059 proteins) were proteins previously detected in the human brain tissue (Supplementary Figure S1C),^32^ with a median of 710 proteins detected in at least 60% of the samples (Supplementary Figure S1D).

For quality control, we observed minimal variation in spiked SUC2 intensities (Supplementary Figure S1E) and consistent iRT peptide elution profiles (Supplementary Figure S1F), thereby confirming adequate sample preparation and LC performance, respectively. The reproducibility of pooled CSF samples (run every 10 samples) was also adequate, with correlations of 0.97 and 0.96 between technical and processing replicates, respectively (Supplementary Figure S1G). These internal controls collectively indicate the high quality and reliability of the data generated by our CSF proteomics pipeline.

### MAIVC Treatment Is Associated with Significant Changes in the CSF Proteome

**Figure 2.**
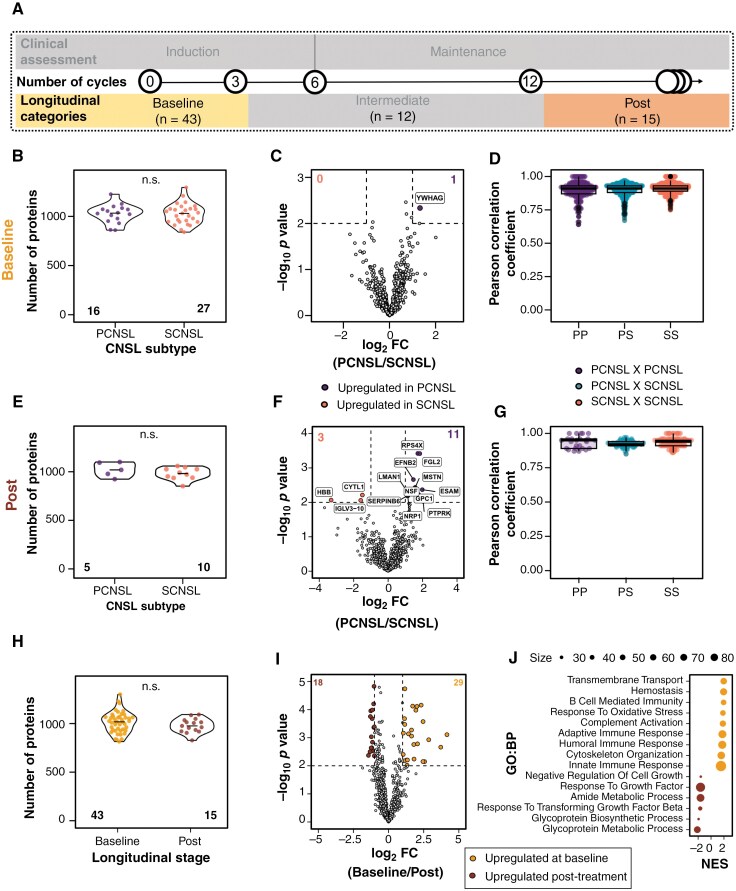
CSF proteome exhibits significant alterations in response to multiagent intraventricular chemotherapy. (A) Longitudinal categories based on the patient’s treatment stage at the time of samples collection [“Baseline” (<3 cycles), “Intermediate” (4-12 cycles), and “Post” (>12 cycles)]. “*n*” represents the number of CSF samples in each category. (B-D) Comparison of PCNSL and SCNSL for “Baseline” samples. (B) Number of proteins detected in each CNSL subtype in “Baseline” samples. *p-*value was computed with unpaired Wilcoxon test. (C) Volcano plot showing the differentially expressed proteins (DEPs; *p* < .01 and |log_2_FC| ≥ 1; unpaired 2-tailed *t*-test) in PCNSL vs SCNSL. (D) Pearson correlation coefficient between (PCNSL and SCNSL) and within proteomic profiles of CNSL subtypes (PCNSL and PCNSL, and SCNSL and SCNSL). (E-G) Comparison of PCNSL and SCNSL in “Post” samples. (E) Number of proteins detected in each CNSL subtype in “Post” samples. *p-*value was computed with unpaired Wilcoxon test. (F) Volcano plot showing the DEPs (*p* < .01 and |log_2_FC| ≥ 1; unpaired 2-tailed *t*-test) in PCNSL vs SCNSL. (G) Pearson correlation coefficient between (PCNSL and SCNSL) and within proteomic profiles of CNSL subtypes (PCNSL and PCNSL, and SCNSL and SCNSL). (H) Number of proteins detected for the 2 timepoints of samples collection (“Baseline” and “Post”). (I) Differentially expressed proteins significance (*p* < .01 and |log_2_FC| ≥ 1; unpaired 2-tailed *t*-test) in “Baseline” vs “Post” samples. Total DEPs are in upper corners. (J) Dot plot of the selected significant terms (FDR < 25%) from GSEA in “Baseline” vs “Post” samples. The size of the dot represents the number of genes involved in the respective pathway. Numbers in bottom corner of boxplots indicate sample size. Total DEPs are in upper corners of the boxplots. PCNSL: primary central nervous system lymphoma; SCNSL: secondary central nervous system lymphoma.

To evaluate for the effects of treatment on the CSF proteomic milieu, CSF samples were categorized into 1 of 3 longitudinal stages (“Baseline” [≤3 cycles], “Intermediate” [4-12 cycles], or “Post” [>12 cycles]) based on the patient’s treatment progress at the time of collection, as illustrated in Figure 2A.

Before we could compare the “Baseline” and “Post” proteomic profiles across all CNSL patients, it was important to determine whether PCNSL and SCNSL samples were sufficiently similar to justify a pooled analysis (Figure 2B-G). We found that the number of proteins detected in PCNSL and SCNSL samples were similar (*p* = .88: “Baseline” [Figure 2B], and *p* = .43: “Post” [Figure 2E]). Differential expression analysis revealed only a handful of proteins that were significantly different between the 2 subtypes (*p* < .01, |log_2_FC| ≥ 1; *t*-test) (Figure 2C, F). Finally, we found that the correlation of proteomic profiles between CNSL subtypes (ie, PCNSL vs SCNSL) was comparable to those within subtypes (ie, PCNSL vs PCNSL, SCNSL vs SCNSL) (Figure 2D, G). Collectively, these analyses suggest that there are no robust proteomic biomarkers that can reliably distinguish between PCNSL and SCNSL patients, within our sample cohort. Based on these findings, we combined data from PCNSL and SCNSL patients for the remainder of our analyses.

To assess the effects of MAIVC treatment on the CSF proteome, we compared “Baseline” and “Post” samples. While quantitatively a similar number of CSF proteins was detected in both groups (*p* = .25, Wilcoxon test) (Figure 2H and Supplementary Figure S2A), differential expression analysis revealed significant differences between “Baseline” and “Post” CSF samples, including 29 upregulated and 18 downregulated proteins at “Baseline” compared with “Post” samples (*p* < .01, |log_2_FC| ≥ 1; *t*-test) (Figure 2I and Supplementary Figure S2B). Using gene set enrichment analysis (GSEA), we found that “Baseline” CSF was associated with greater degrees of oxidative stress and immune-related signaling, whereas “Post” CSF samples were enriched for proteins involved in cell growth regulation, amide metabolism, and glycoprotein metabolism (Figure 2J). While pathway analysis was conducted on a selected set of proteins from biofluids and thus may not capture all relevant pathway components, these results suggest potential BP worthy of future investigation, rather than definitive mechanistic conclusions. In conclusion, our findings indicate that the CSF proteome in CNSL patients is dynamic and changes in response to MAIVC therapy.

### The CSF Proteome Can Be Leveraged to Classify Clearance of CSF in Response to MAIVC

**Figure 3.**
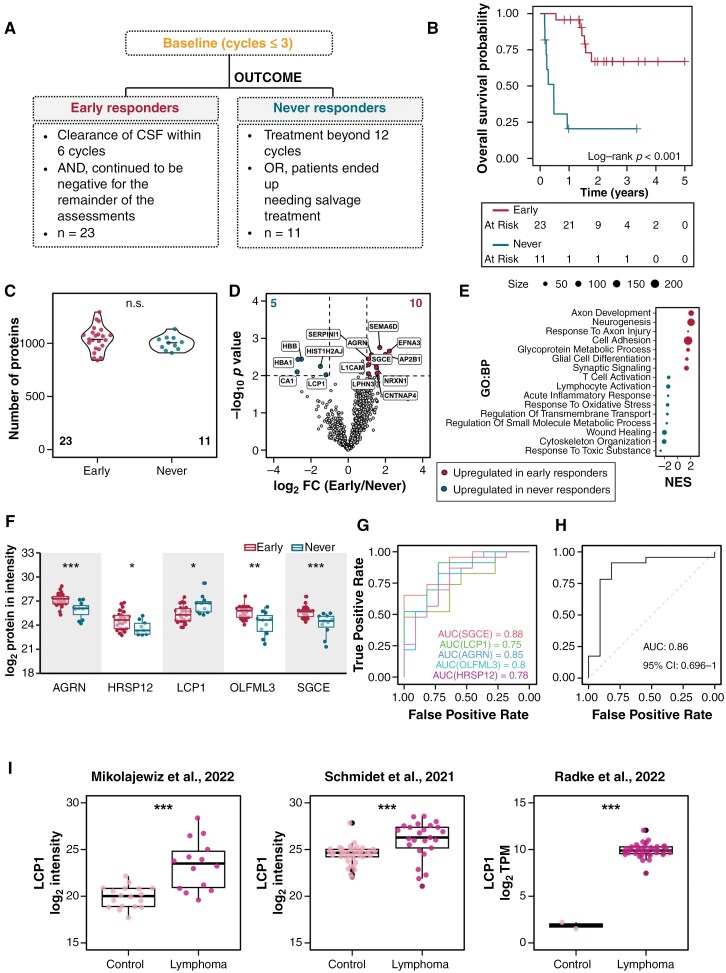
“Baseline” CSF protein signature distinguishes “Early” and “Never” responders to MAIVC. (A) Flowchart depicting the criteria for response assessment. “Baseline” samples of “Early” and “Never” responders were considered to identify prognostic markers to MAIVC. (B) Kaplan-Meier survival curve of the patients stratified by response to MAIVC therapy. “Early” responders perform significantly better than “Never” responders (*p* < .001, log-rank test). (C) Number of proteins detected in each of the response groups. (D) Volcano plot visualizing the DEPs between “Early” vs “Never” responders. Dashed lines represent the threshold for significance (*p* < .01 and |log_2_FC| ≥ 1; unpaired 2-tailed *t*-test). (E) Dot plot of the selected significant terms (FDR < 25%) in pathway enrichment analysis performed with GSEA. The size of the dot represents the number of genes involved in the respective pathway. (F) Box plot showing the log_2_ protein intensities of key features distinguishing “Early” and “Never” responders identified from machine learning algorithm. (G) Comparison of the ROC curves of the candidate proteins in classifying “Early” vs “Never” responders. Individual models were trained with logistic regression and internally validated with LOOCV. (H) ROC curve of the combined performance of the 5 candidate proteins trained with logistic regression and internally validated with LOOCV. (I) Expression of LCP1 in three external independent cohorts comparing lymphoma vs control. Mikolajewicz and Schmid cohort were CSF proteomes, where Radke cohort is tissue RNAseq. Significance was determined as *p*-value < .05*, < .01**, or < .001*. Numbers in bottom corner indicate sample size. Total DEPs are in upper corners. FDR: false discovery rate; ROC: Receiver-operating characteristic curve; LOOCV: leave-one-out-cross-validation; AUC: area under curve; CI: confidence interval.

We next sought to determine whether the CSF proteome can be used to classify response to MAIVC. We compared patients with an early response to treatment (ie, negative CSF cytology achieved within 6 treatment cycles, and subsequently sustained) with those that never responded to treatment (ie, CSF cytology positive beyond 12 cycles or required salvage treatment; Figure 3A and Supplementary Figure S3A). As expected, “Early” responders had favorable survival outcomes compared with “Never” responders (Figure 3B; *p* < .001, Log-rank test). Notably, the age distribution (Supplementary Figure S3B; *p* = .48, Wilcoxon test) and number of proteins detected in each response groups was similar (Figure 3C; *p* = .27, Wilcoxon test). Differential expression analysis between the 2 groups identified 10 upregulated and 5 downregulated proteins in “Early” responders (Figure 3D and Supplementary Figure S3C; *p* < .01 and |log_2_FC| ≥ 1; *t*-test). GSEA further revealed that the CSF proteome of “Early” responders was enriched for proteins involved in axon development and neurogenesis, whereas the CSF of “Never” responders was enriched for T cell activation oxidative stress (Figure 3E).

Next, we used a machine learning approach to classify patients based on their response to MAIVC using “Baseline” CSF proteomics. Regularization-based feature selection identified candidate biomarkers capable of discriminating between “Early” and “Never” responders: SGCE, LCP1, AGRN, OLFML3, and HRSP12 (Figure 3F and Supplementary Figure S3D-E, see Methods). LCP1 was the only candidate biomarker significantly upregulated in “Never” responders compared with “Early” responders, whereas all other biomarkers were downregulated (Figure 3F). Notably, Kaplan-Meier analysis of overall survival stratified by LCP1 expression levels showed a trend toward poorer survival in patients with high LCP1 levels (Supplementary Figure S3H). The AUROC performance of individual candidates in classifying “Early” versus “Never” responders, determined using logistic regression, ranged from 0.75 (95% CI: 0.58-0.92) for LCP1 to 0.88 (95% CI: 0.77-1.0) for SGCE (Figure 3G). Interestingly, the AUROC performance did not improve with models trained with logistic regression using all 5 candidate proteins (0.88 [95% CI: 0.74-1.0]), suggesting redundant information across the 5 biomarkers (Figure 3H and Supplementary Figure S3F).

Finally, to explore the specificity of these candidates, we analyzed expression in tumor versus control samples using 3 independent external cohorts, including the Schmid cohort (CSF proteome),^27^ Mikolajewicz cohort (CSF proteome),^26^ and Radke cohort (tissue RNA-seq)^33^ (Supplementary Figure S3G). LCP1 was the only biomarker that was significantly elevated in CNSL samples across all cohorts (Figure 3I). We conclude that LCP1 is a CNSL-enriched biomarker that can be used to classify patient CSF disease clearance in response to MAIVC treatment using “Baseline” CSF samples.

### CSF Tumor Burden Can Be Monitored using CSF Proteomic Biomarkers

**Figure 4.**
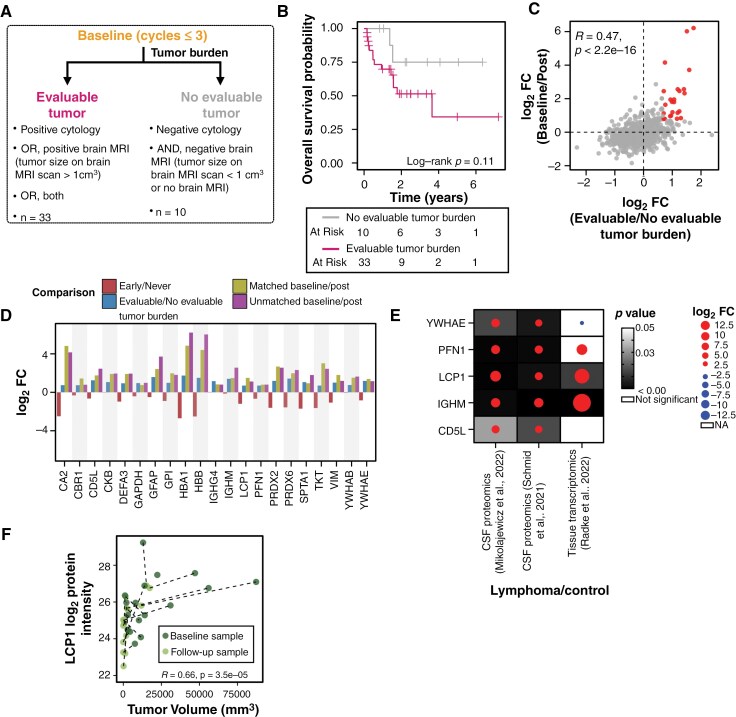
Protein biomarker can complement current diagnostic approaches. (A) Flowchart for classifying “Baseline” samples of patients based on “Evaluable” or “No evaluable” tumor burden. (B) Kaplan-Meier survival curve of patients stratified by tumor burden at “Baseline.” (C) Scatter plot of effect sizes between treatment stages (“Baseline”/“Post”) and tumor burden (“Evaluable”/“No evaluable”). Highlighted proteins have log_2_FC > 0.7 for both comparisons. Correlation coefficient was computed with Pearson correlation test. (D)Bar plot of the 21 candidate proteins showing the log_2_FC for varied comparisons performed in this study: “Early” vs “Never” responders at “Baseline”; “Evaluable tumor burden” vs “No evaluable tumor burden” at “Baseline”; matched “Baseline” and “Post” samples; and unmatched “Baseline” and “Post” samples. (E) Dot plot showing the expression of 5 putative tumor burden markers in the three external cohorts comparing CNSL and control samples: Mikolajewicz cohort (CSF proteome), Schmid cohort (CSF proteome), and Radke cohort (Tissue RNAseq). Size of the dot corresponds to the effect size difference and the background shading is a measure of its significance computed with unpaired 2-tailed *t*-test. (F) Correlation of LCP1 expression in the “Baseline” and the follow-up sample of patients with “Evaluable tumor burden” at “Baseline.” Tumor volume (mm^3^) was calculated from craniospinal MRI imaging. Correlation coefficient was computed with Spearman correlation test.

To identify tumor burden biomarkers in CSF, we compared the proteome of patients with “Evaluable tumor burden” and “No evaluable tumor burden.” (Figure 4A). As expected, patients with “No evaluable tumor burden” at “Baseline” showed a tendency toward favorable overall survival compared with patients with “Evaluable tumor burden” disease (*p* = .11, Log-rank test) (Figure 4B).

A tumor burden marker should serve as a diagnostic (ie, detect tumor at diagnosis) and surveillance/monitoring (eg, monitor changes in tumor burden in response to treatment) tool. We hypothesized that such a marker would be elevated in patients with “Evaluable tumor burden” at baseline and should downregulate as a response to treatment. To test this, we performed differential expression analyses between “Baseline” versus “Post” samples and between “Evaluable tumor burden” versus “No evaluable tumor burden” samples, and compared the resulting log_2_FC values (Figure 4C). To minimize risk of aggregation bias, we also performed paired analyses across the 7 matched “Baseline”/“Post” longitudinal samples (Supplementary Figure S4A). The intersection of these results (Supplementary Figure S4B) identified 21 proteins of interest (Figure 4D). Of these 21, we focused on 5 proteins (YWHAE, PFN1, LCP1, IGHM, CD5L) that were enriched in external CNSL cohorts,^26,27,33^ thereby supporting their utility as CNSL-enriched tumor burden biomarkers (Figure 4E and Supplementary Figure S4C).

Our analysis revealed that LCP1, which was associated with CSF response to MAIVC (Figure 3G), also satisfied our CSF tumor burden biomarker criteria. As anticipated, we found that MAIVC “Never”-responders tended to have higher tumor burden (Supplementary Figure S4D). As one would expect, this suggests that the efficacy of MAIVC may be limited in patients with larger tumors, due to limited drug penetrance and exposure through the tumor mass. This highlights the importance of considering both parenchymal and CSF tumor burden in treatment planning.

Among the patients in whom tumor burden could be estimated by MRI and CSF biomarkers, the correlation between LCP1 levels and tumor volume was 0.66 (*p* = 3.5e−05, Spearman correlation test; Figure 4F). Notably, LCP1 levels had a greater dynamic readout for smaller tumors (<9318 mm^3^), suggesting that it could be used to detect small changes in even in parenchymal tumor volume (Figure 4F). In patients in which tumors were evaluable by only 1 modality (ie, CSF or MRI), LCP1 levels were consistently elevated, reflecting the superior sensitivity of CSF proteomics in monitoring tumor burden in general. This opens opportunities to detect MRD earlier, thereby avoiding delays in treatment (Supplementary Figure S4E).

## Discussion

In this study, we conducted a comprehensive analysis of CSF proteomic profiles from 59 CNSL patients undergoing the MAIVC treatment regimen. Using the MStern sample processing approach, we identified 2059 proteins across 115 CSF samples.

We observed considerable overlap in the CSF proteome of PCNSL and SCNSL at both “Baseline” and “Post” time points, which aligns with the current body of literature, as no other studies have identified reliable protein markers to distinguish these subtypes, possibly due to the clonal evolution of tumor cells that leads to convergent protein expression patterns across different lymphoma origins as they infiltrate the CNS.^34^ While sporadic research has suggested potential differentiation using miRNA markers, these findings have not been thoroughly validated.^35,36^ We observed distinct baseline proteomic signatures distinguishing between “Early” treatment responders and “Never”-responders. We identified and externally validated LCP1 as significantly elevated in CNSL samples, particularly in “Never”-responders. However, it remains unclear whether these proteomic changes are directly attributable to therapeutic intervention or the reduction in tumor mass. We also identified YWHAE, PFN1, LCP1, IGHM, and CD5L as potential tumor burden markers. To assess their reliability, we evaluated the coefficient of variation of these markers in the cohort (Supplementary Figure S5A-E). Most markers demonstrated good analytical stability with CVs below 10%. These candidates were found to be enriched in external CNSL cohorts, supporting their utility as CNSL-enriched tumor burden markers.

One major finding of our study was the identification of LCP1 (lymphocyte cytosolic protein 1), also known as plastin-2, as a candidate predictive marker for MAIVC efficacy and disease monitoring in CNSL. LCP1 is expressed in hematopoietic cell lineages and various cancers, where it regulates actin dynamics and cellular adhesion, facilitating tumor progression.^37–39^ While its role has been established in other hematologic malignancies such as chronic lymphocytic leukemia,^40^ direct evidence of its production by CNSL cells remains to be demonstrated. Given that LCP1 is involved in immune cell function, its elevation in CSF could potentially reflect other conditions with CNS inflammation or lymphocytic infiltration. Future studies examining LCP1 expression in CNSL cells and comparing its levels across different CNS pathologies are essential to establish its specificity as a biomarker. Additionally, larger prospective clinical cohorts will be needed to evaluate its potential as a monitoring tool for disease progression, which we plan to pursue in future studies.

Our study highlights the diminished efficacy of MAIVC in patients with high parenchymal tumor burden, underscoring the importance of integrating both parenchymal and CSF tumor burden assessment into treatment planning. Observations akin to our findings have been documented in various lymphoma types, such as diffuse large B-cell lymphoma (DLBCL),^41,42^ follicular lymphoma (FL),^43^ Burkitt lymphoma,^44^ and primary cutaneous T-cell lymphoma.^45^ The significance of overall tumor burden has also been established in numerous solid tumors, including non-small-cell lung cancer,^46^ breast cancer,^47,48^ and metastatic melanoma.^49^ It is important to note that MAIVC is designed primarily as a compartmental therapy for treating CSF-based disease, although it demonstrates some beneficial effects on parenchymal tumors. Given its limited efficacy in patients with higher parenchymal tumor burden, it remains unclear whether this reduced efficacy is primarily attributable to the location of the tumor (intraparenchymal vs CSF) or to the overall volume of the tumor, regardless of its location. Future studies may benefit from a more nuanced approach, differentiating between intraparenchymal and CSF tumor burdens to provide clearer insights into the specific efficacy of MAIVC in each compartment. While the potential tumor burden markers (YWHAE, PFN1, LCP1, IGHM, CD5L) are relatively abundant in CSF and have been previously implicated in various malignancies beyond CNSL, our study uniquely demonstrates their value in monitoring CNSL disease progression and treatment response. Specifically, we propose that the dynamic changes in these proteins during treatment, rather than their absolute presence, could serve as useful markers for monitoring CNSL. Future comparative studies across different CNS pathologies will be valuable to further define the specificity of these temporal patterns in CNSL.

Our study findings should be interpreted with several caveats. The dual role of LCP1 as both a biomarker for MAIVC response and a tumor burden marker necessitates additional validation through prospective studies conducted in a more standardized manner. In analyzing biomarkers of response, we acknowledge that pairwise comparison of samples from the same individual would provide better control for interindividual proteomic variability than our group comparison approach. Although our study design precluded paired analysis for all samples, we demonstrated temporal LCP1 changes within individual patients through paired analysis of matched “Baseline”—“Post” samples in a subset of 4 “Early” responders (Supplementary Figure S5F).

While our CSF-directed therapy was part of a standardized protocol, the overall heterogeneity of the patient cohort and potential confounders such as variations in systemic therapy and radiation therapy may have influenced our results. Given the rare nature of CNSL and our exploratory focus, we addressed the challenges of small sample size by prioritizing proteins with substantial effect sizes. While we did not apply strict FDR correction, the biological significance of our findings is supported by multiple validation approaches, including leave-one-out cross-validation to address potential overfitting, external cohort verification to demonstrate reproducibility, and pathway analyses to establish biological relevance.

Even though protein depletion protocols can theoretically improve the detection of low-abundance proteins, it can potentially introduce variability and inadvertently affect the detection of bound low-abundance proteins through protein–protein interactions. Our approach effectively captured meaningful proteomic changes as evidenced by our quality control metrics and findings.

While our findings highlight LCP1’s potential in treatment response classification, understanding its direct expression in CNSL cells would provide mechanistic insights and strengthen its clinical application for tracking treatment outcomes. Future comparative studies across different CNS pathologies will be valuable to further define the specificity of these temporal patterns in CNSL and establish LCP1’s specificity as a CNSL biomarker. Additionally, the development of specialized, high-sensitivity assays, such as Parallel Reaction Monitoring and Enzyme-Linked Immunosorbent Assay, will be crucial to accurately quantify LCP1 levels within clinical samples.

## Supplementary Material

vdaf046_suppl_Supplementary_Figures_S1-S5

vdaf046_suppl_Supplementary_Tables_S1

vdaf046_suppl_Supplementary_Tables_S2

vdaf046_suppl_Supplementary_Tables_S3

vdaf046_suppl_Supplementary_Tables_S4

vdaf046_suppl_Supplementary_Tables_S5

## Data Availability

Proteomic data generated in this study have been deposited to MassIVE (identifier: MSV000095917). Proteomic data from Mikolajewicz et al.^26^ were obtained from MassIVE (MSV000089062) and Schmid et al.^27^ from ProteomeXchange (PXD021984). RNA-seq data reported by Radke et al.^33^ was obtained from European Genome-Phenome Archive (EGAS00001005339).
