# Supplementary material for: Cerebrospinal fluid protein biomarkers are associated with response to multiagent intraventricular chemotherapy in patients with CNS lymphoma
Source: Neurooncol Adv. 2025 Feb 25;7(1):vdaf046. doi: 10.1093/noajnl/vdaf046 (PMC12048878; doi:10.1093/noajnl/vdaf046)
Supplement: vdaf046_suppl_Supplementary_Figures_S1-S5 [file vdaf046_suppl_supplementary_figures_s1-s5.docx]

**Supplementary figures**

**
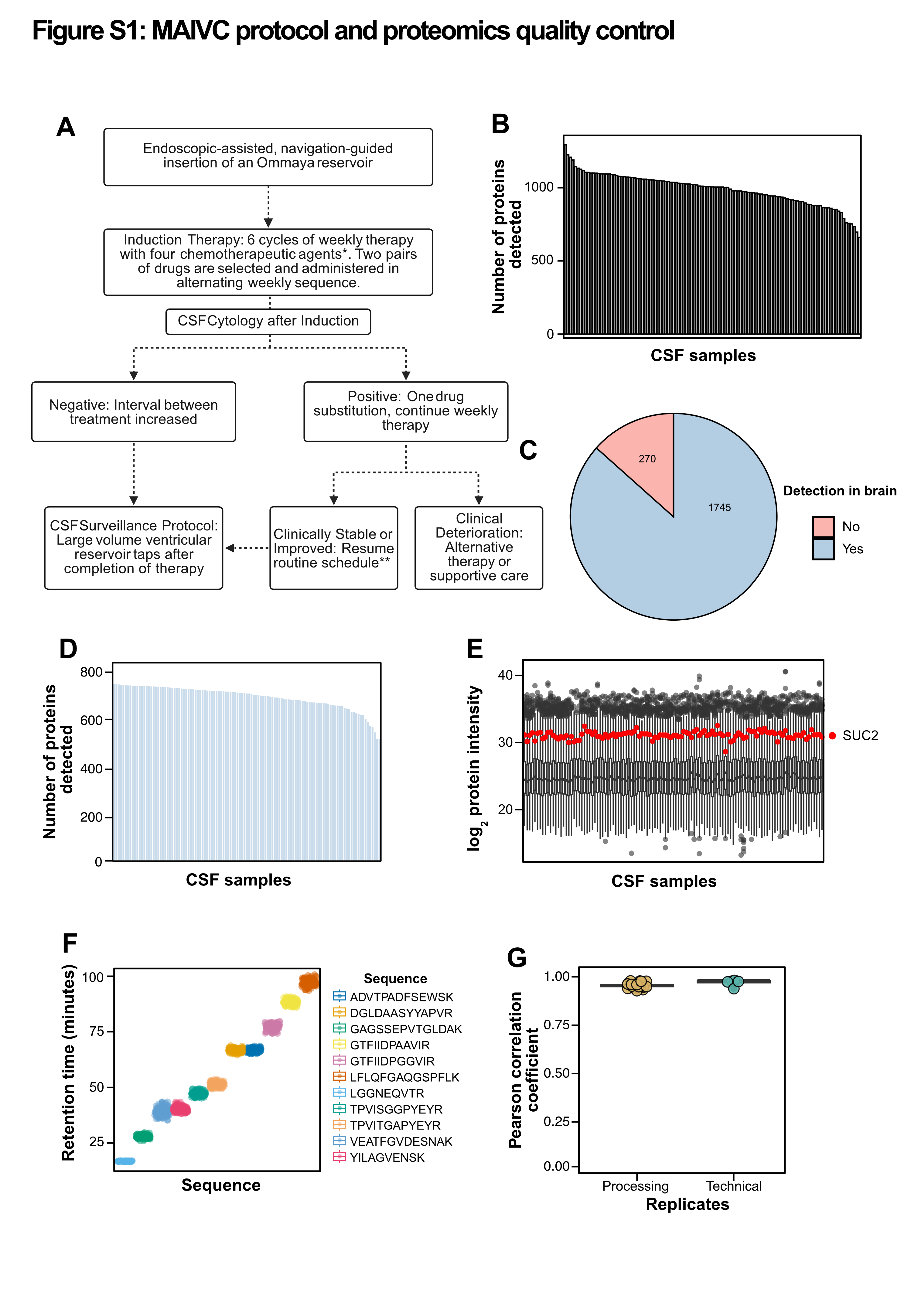
**

**Figure Supplement 1. Quality Control**

**(A)** Treatment protocol for MAIVC treatment. *Eight possible chemotherapeutic agents: methotrexate, thiotepa, etoposide, topotecan, liposomal cytarabine, cytarabine, gemcitabine, rituximab. **Routine schedule is considered an increase in the interval between MAIVC treatments: 2 cycles of biweekly therapy, 2 cycles of tri-weekly therapy, and 2 cycles of quatri-weekly therapy.

**(B)** Boxplot showing the number of proteins detected in each sample.

**(C)** Number of brain-enriched proteins detected in the cohort.

**(D)** Boxplot depicting the detection of brain-enriched proteins in CSF samples. The plot shows the percentage of proteins identified in at least 60% of the samples. Each bar represents an individual sample.

**(E)** Boxplot showing the log_2_ protein intensities along with the SUC2 intensities in each sample. **(F)** Average retention time of iRT peptides across all samples.

**(G)** Pearson correlation coefficient between pooled CSF QC samples ran as processing and technical replicates.

iRT: indexed retention time.


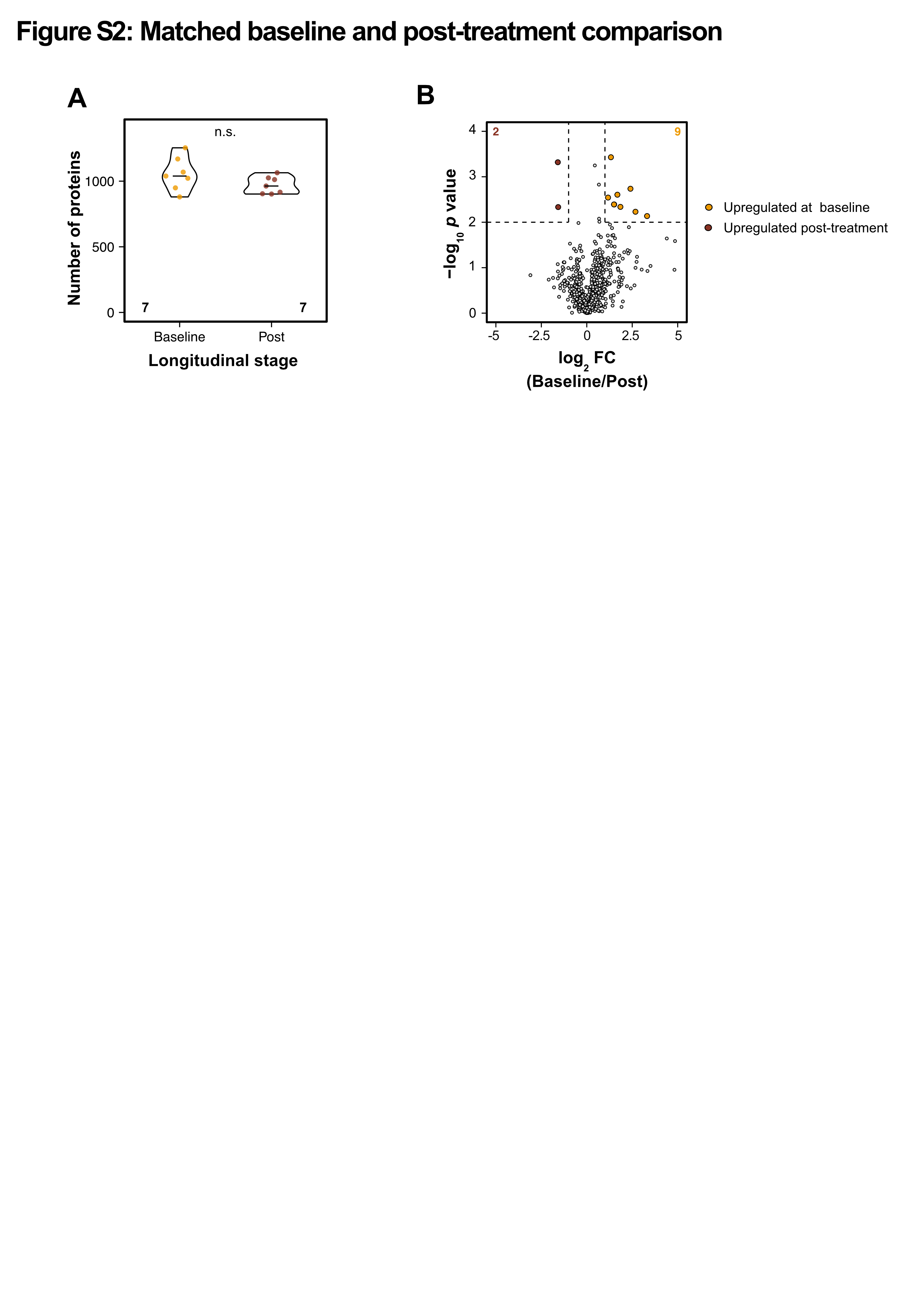


**Figure Supplement 2. Matched “Baseline” and “Post” comparison**

**(A)** Number of proteins detected in each group. *p* value was computed with Wilcoxon test.

**(B)** Volcano plot showing the differentially expressed proteins between the two temporal stages of treatment of matched samples. Dashed lines represent the threshold for significance (*p* < 0.01 and |log_2_FC| ≥ 1; unpaired two-tailed t-test).

Numbers in bottom corner indicate sample size. Total differentially expressed proteins are in upper corners.

**
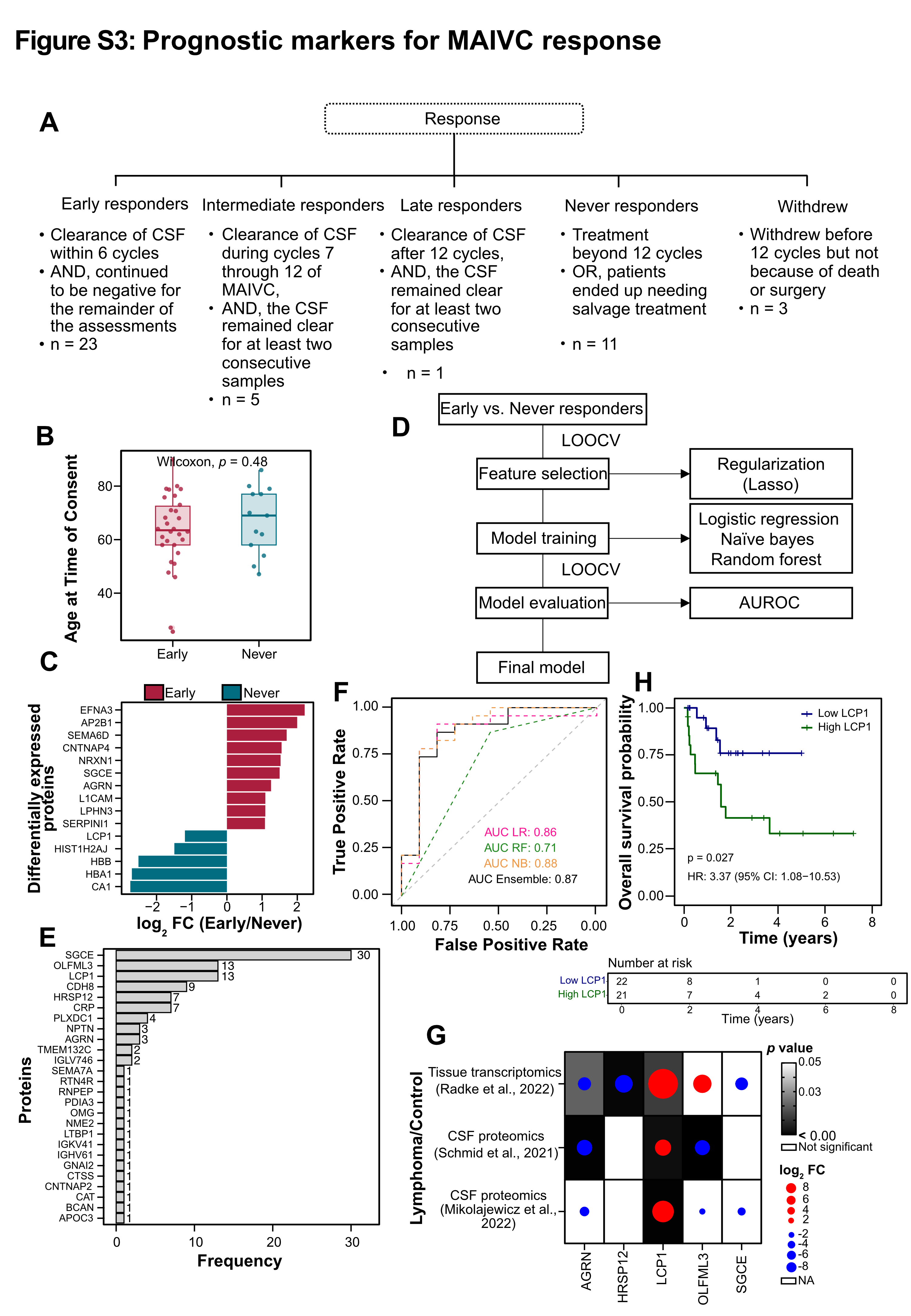
**

**Figure Supplement 3. Prognostic markers for MAIVC response**

**(A)** Flowchart depicting the response assessment criteria for MAIVC. Samples were characterised at “Baseline”, where ‘n’ represents the number of samples in each category.

**(B)** Boxplot showing the comparison of age of patients in each response group. *p* value was computed with unpaired Wilcoxon test.

**(C)** Waterfall plot showing the effect size difference of the differentially expressed proteins in “Baseline” samples of “Early” vs. “Never” responders.

**(D)** Machine learning workflow to identify prognostic markers for classifying “Early” and “Never responders.

**(E)** Histogram depicting the frequency of each protein in classifying “Early” and “Never responders, with candidate proteins on the X-axis and the number of models on the Y-axis.

**(F)** ROC curves of the trained models by combining the 5 candidate proteins as one protein signature.

LOOCV: leave one out cross validation; AUROC: area under receiver operating curve; LR: logistic regression; RF: random forest and NB: naïve bayes.

**(G)** Dot plot showing the expression of the five candidate markers in the three external independent cohorts comparing CNSL and control samples: Mikolajewicz cohort (CSF proteome), Schmid cohort (CSF proteome) and Radke cohort (Tissue RNAseq). Size of the dot corresponds to the effect size difference and the background shading is a measure of its significance computed with unpaired two-tailed t-test.

**(H)** Kaplan-Meier survival curve of patients stratified by median LCP1 expression at “Baseline” in our cohort.


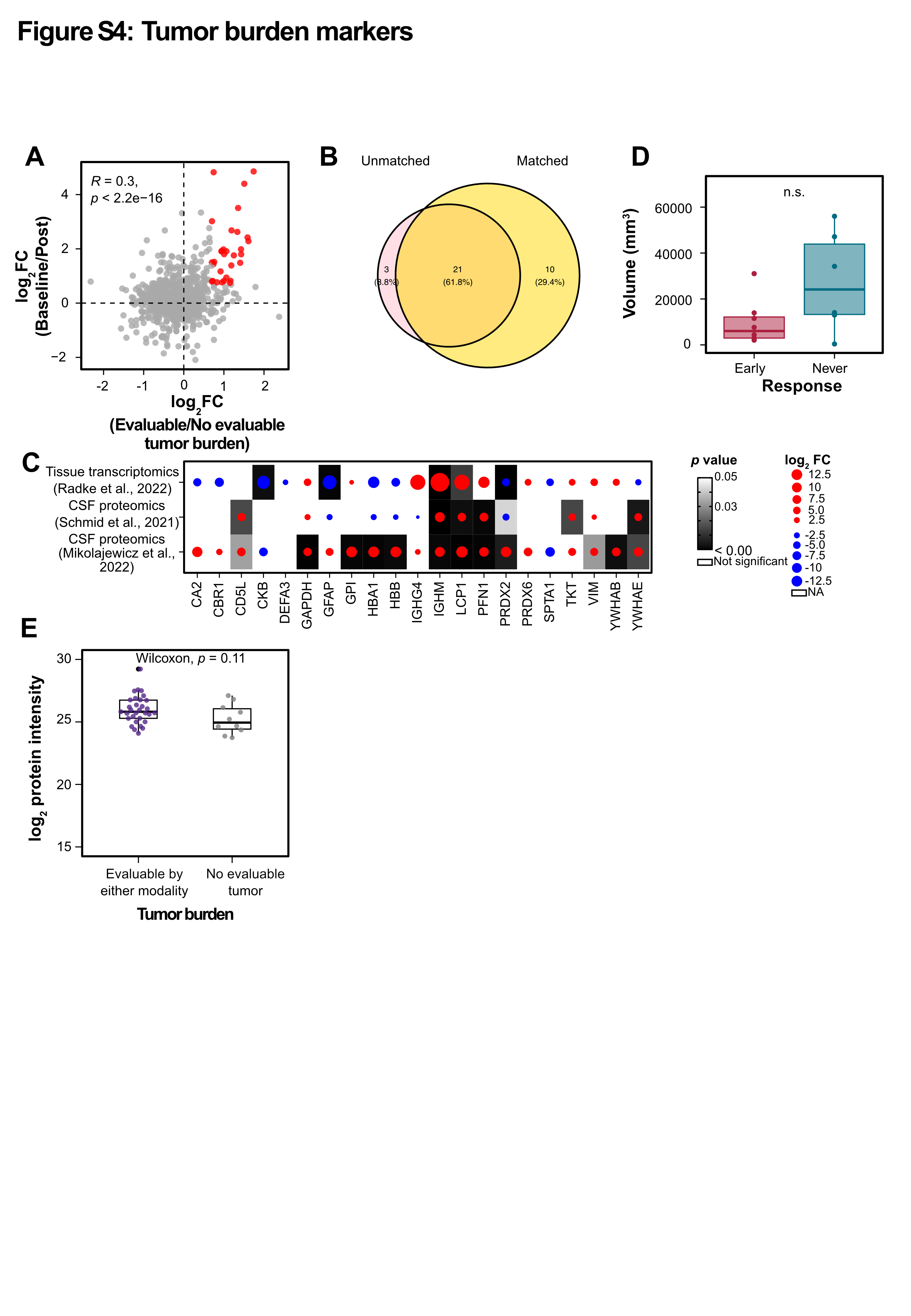


**Figure Supplement 4. Tumor burden markers**

**(A)** Two-way scatter plot of effect size difference between treatment stages for matched samples (“Baseline”/“Post”) and tumor burden at “Baseline” (“Evaluable”/“No evaluable” tumor). Highlighted proteins have log_2_FC > 0.7 for both comparisons. Correlation coefficient was computed with Pearson correlation test.

**(B)** Venn diagram showing the intersection of the highlighted proteins in Fig 4C and S4A.

**(C)** Dot plot showing the expression of 21 proteins of interest in the three external independent cohorts comparing CNSL and control samples: Mikolajewicz cohort (CSF proteome), Schmid cohort (CSF proteome) and Radke cohort (Tissue RNAseq). Size of the dot corresponds to the effect size difference and the background shading is a measure of its significance computed with unpaired two-tailed t-test.

**(D)** Boxplot showing the tumor volume calculated with MRI brain scans at “Baseline” for “Early” and “Never” responders. *p* value was computed with Wilcoxon test.

**(E)** Relative abundance of LCP1 in tumors evaluable by only one modality (MRI or CSF cytology) compared to “No evaluable tumor burden”. *p* value was computed with unpaired Wilcoxon test.

**
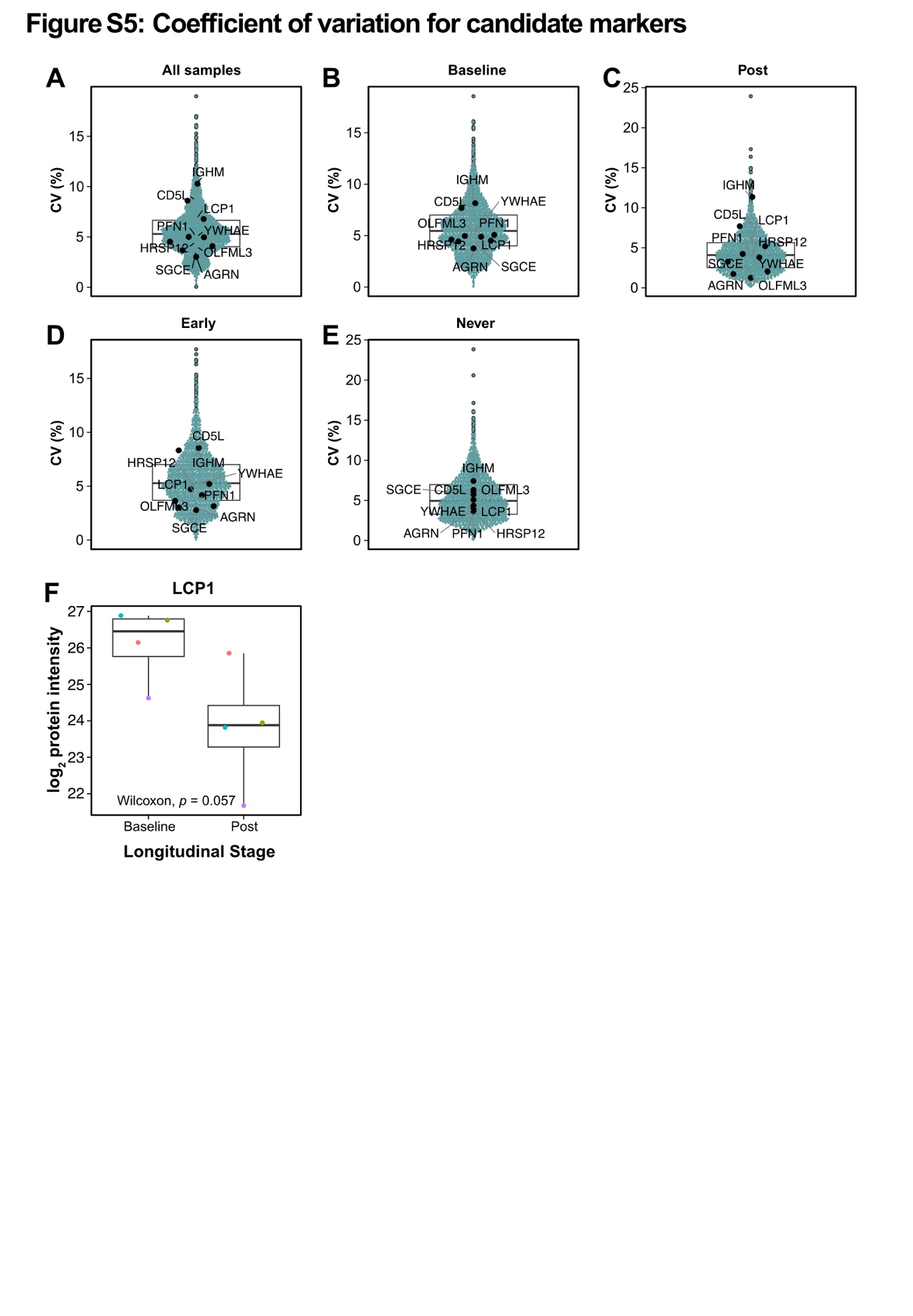
**

**Figure Supplement 5. Candidate markers**

**(A-E) Analysis of protein abundance variability across sample groups. Coefficient of variation (CV) shown as percentages for log_2_-transformed protein abundance data. Analysis includes: (A) All samples (B) “Baseline” samples only (C) “Post” samples only (D) “Baseline” samples from “Early” responders (E) “Baseline” samples from “Never”-responders.**

**(F) LCP1 protein expression levels compared between 4 matched “Baseline” and “Post” samples.**

Supplementary tables

Table S1. Combination MAIVC regimen for patients with CNS Lymphoma

Table S2. Clinical characteristics of study participants.

Table S3. Processed proteomic profiles of CSF samples.

Table S4. Results from pathway analysis, related to figure 2J.

Table S5. Results from pathway analysis, related to figure 3E.
