# Supplementary material for: Cerebrospinal fluid protein biomarkers are associated with response to multiagent intraventricular chemotherapy in patients with CNS lymphoma
Source: Neurooncol Adv. 2025 Feb 25;7(1):vdaf046. doi: 10.1093/noajnl/vdaf046 (PMC12048878; doi:10.1093/noajnl/vdaf046)
Supplement: vdaf046_suppl_Supplementary_Tables_S1 [file vdaf046_suppl_supplementary_tables_s1.docx]

| Medication |  |
| --- | --- |
| Methotrexate  (15 mg) | C |
| ThioTEPA  (15 mg) | A |
| Etoposide  (2 mg) | B |
| Topotecan  (0.4 mg) | C |
| Liposomal Cytarabine (50 mg) | C |
| Cytarabine (100 mg) | B |
| Gemcitabine  (5 mg) | A |
| Rituximab  (25 mg) | C |

**Table S1**: Combination Multi-Agent Intraventricular Chemotherapy Regimen for Patients with CNS Lymphoma

Two-drug combinations (A-A and B-B) are administered on a fixed, alternating schedule (see text). C represents alternative agents in the setting of drug intolerance, inadequate response, or specific molecular features of the tumor.
